# Talonavicular Arthrodesis Using a Screw and Compression Staple in a Patient With Bipartite Navicular Bone: A Case Report

**DOI:** 10.7759/cureus.43122

**Published:** 2023-08-08

**Authors:** Taro Chujo, Tomoyuki Nakasa, Yasunari Ikuta, Shingo Kawabata, Nobuo Adachi

**Affiliations:** 1 Department of Orthopaedic Surgery, Graduate School of Biomedical and Health Sciences, Hiroshima University, Hiroshima, JPN

**Keywords:** nonunion, compression staple, arthrodesis, talonavicular joint, bipartite navicular

## Abstract

The bipartite navicular bone is a relatively rare pathological condition in which the navicular bone is segmented. As a high nonunion rate in talonavicular arthrodesis of the foot has been reported, an effective fixation method is required to achieve bone union. A compression staple can provide a persistent strong compressive force on the bone surface, which is advantageous for arthrodesis, especially for the joints with a high incidence of nonunion. A 13-year-old boy presenting with left foot pain was diagnosed with bipartite navicular. Imaging of the left foot showed that the navicular bone was divided into two parts and flatfoot deformity. After the failure of conservative treatment, talonavicular arthrodesis was performed. The lateral fragment was removed, and the talar and medial fragments were fixed using a cannulated cancellous screw (CCS) (Ace Medical, El Segundo, CA, USA) and compression staple (DynaNite, 15 mm × 12 mm, Arthrex, Inc., Naples, USA) to correct the flatfoot. Bone union was achieved, and flatfoot improved. Thirteen months postoperatively, his symptoms disappeared, and all categories of the Self-Administered Foot Evaluation Questionnaire scored 100 points. Although the bipartite navicular bone has no established treatment due to its rareness, talonavicular arthrodesis using a combination of CCS and compression staple yields good short-term clinical outcomes including good alignment.

## Introduction

Bipartite navicular is a relatively rare pathological condition [1], characterized by segmentation of the navicular into two or three parts, and typically presents with patients complaining of chronic pain at the dorsal medial aspect and a flatfoot deformity [2]. It has been reported that bipartite navicular bone is a cause of Mueller-Weiss disease, which causes spontaneous osteonecrosis of the tarsal navicular joint [3]. Due to the poor blood supply and bone quality of the navicular bone, internal fixation to restore the motion and function of the midfoot joints often results in nonunion [3-5]. Therefore, current operative procedures involve medial column arthrodesis for the late stage of the bipartite navicular bone [6,7]. Talonavicular arthrodesis has been widely performed for the reconstruction of the medial column and can eliminate pain by joint fixation. Although talonavicular arthrodesis of the foot is an effective procedure for improving symptoms and function, a high nonunion rate has been reported, ranging from 3% to 37% [8]. Additionally, there are few reports of talonavicular arthrodesis in bipartite navicular, and there is no consensus on the optimal fixation method for talonavicular arthrodesis. Therefore, an effective fixation method that increases the efficacy of bone union is required. Recently, compression staples have become widely used in foot and ankle surgery [9]. They have a compression ability due to the material properties of superelasticity and shape memory and can be used in a small space, unlike the use of surgical plates [9,10]. We report a case of bipartite navicular surgery in which good clinical outcomes were obtained by talonavicular arthrodesis using a compression staple and screw.

## Case presentation

**Figure 1:**
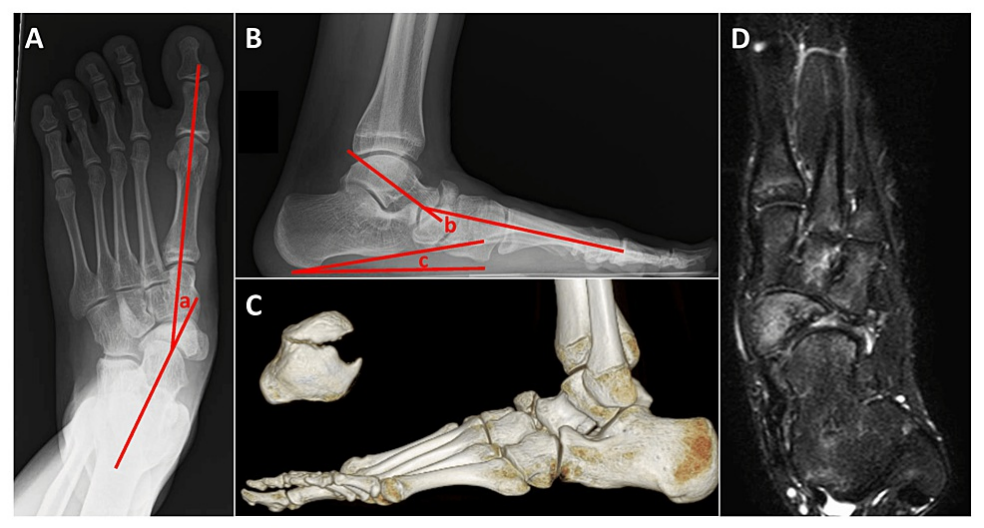
Imaging of the bipartite navicular (A) Anteroposterior view of the weight-bearing radiogram; (B) Lateral view of the weight-bearing radiogram; (C) Computed tomography (CT); (D) T2-weighted fat-suppressed magnetic resonance imaging. a, b: talo-first metatarsal angle; c: calcaneal pitch

A 13-year-old male had left foot pain for two years without any specific cause. He was treated conservatively with arch support at a local orthopaedic clinic; however, his pain, especially while playing basketball, did not improve. The patient was referred to our hospital for further treatment. Physical examination revealed a flat foot and protrusion of the dorsal aspect of the left foot. Tenderness of the left navicular bone was observed, and the ranges of motion were as follows; dorsiflexion 25°; plantar flexion 45°; inversion 30°; and eversion 35° on the left, and dorsiflexion 25°; plantar flexion 45°; inversion 35°; and eversion 35° on the right. Plain radiographs on weight-bearing showed the navicular bone separated into two parts and a lateral fragment displaced to the dorsal side. A flat foot deformity with a talo-first metatarsal angle of 28° on the anteroposterior radiograph, a talo-first metatarsal angle of 35°, and a calcaneal pitch of 8° on the lateral radiograph were also observed. Irregularity in the joint space of the talonavicular and naviculocuneiform joints was also observed (Figure 1A-1B). Computed tomography (CT) showed that the dorsal fragment was displaced dorsally, and the plantar fragment was shifted to the plantar and medial sides. Osteophyte formation was also observed around the talonavicular joint (Figure 1C). On magnetic resonance imaging (MRI), bone marrow lesions were observed in the entire navicular region on T2-weighted images (Figure 1D).

**Figure 2:**
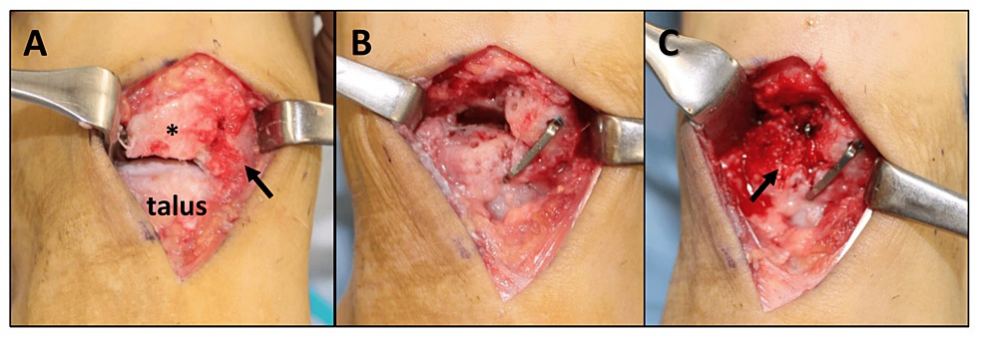
Operative findings (A) Exposing the talonavicular joint. *: Lateral fragment. Arrow: medial fragment of the navicular. (B) After fixation of the talonavicular joint. (C) Bone grafting (arrow).

The Japanese Society for Surgery of the Foot (JSSF) midfoot scale score was 60 [11]. The Self-Administered Foot Evaluation Questionnaire (SAFE-Q) scores were 55.0 for pain, 72.7 for physical function, 54.2 for social function, 91.7 for shoe-related, 85.0 for general health, and 44.4 for sports [12]. We diagnosed his condition as bipartite navicular bone with osteoarthritic changes and performed talonavicular joint arthrodesis to treat his condition. A longitudinal skin incision 5cm in length was made on the talonavicular joint and the navicular bone was exposed. The navicular bone was segmented into fibrously adhered lateral and medial fragments. The lateral fragment was removed and the talonavicular joint was exposed. In the talonavicular joint, the articular cartilage was extensively lost, and the subchondral bone was exposed (Figure 2A). The medial fragment was displaced to the plantar and medial sides. The sclerotic subchondral bone of the talonavicular joint was excised. The medial fragment was lifted and lateralized so that a longitudinal arch was formed and then, the talonavicular joint was fixed using a 4.0mm diameter of cannulated cancellous screw (CCS) (Ace Medical, El Segundo, CA, USA). A compression staple (DynaNite, 15 mm × 12 mm, Arthrex, Inc., Naples, USA) was used to fix the joint firmly (Figure 2B). The cancellous bone was harvested from the medial malleolus and transplanted into a bone defect in the talonavicular joint (Figure 2C).

**Figure 3:**
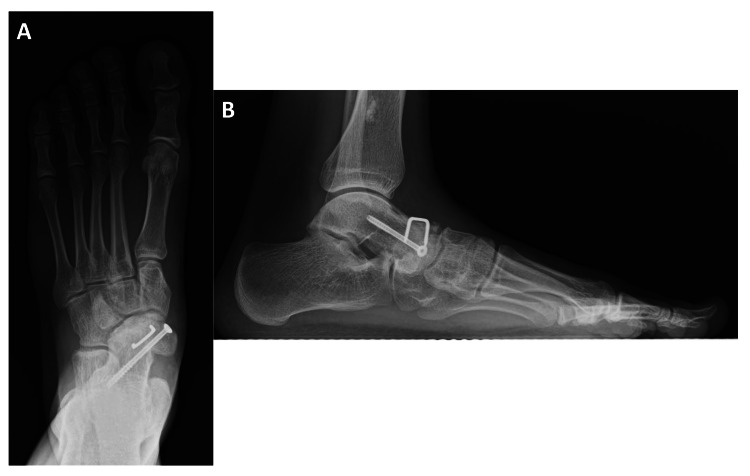
Plain radiograms with weight-bearing at eight months after surgery. (A) Anteroposterior view. (B) Lateral view

A short-leg cast was applied for five weeks. A rubber heel was attached to the cast, and weight-bearing was permitted two weeks postoperatively. During the postoperative course, his symptoms completely abated, and the bone union was confirmed on radiography three months postoperatively. He was able to return to playing basketball six months post-surgery. Eight months after surgery, the CCS and a compression staple were removed (Figure 3A-3B).

**Figure 4:**
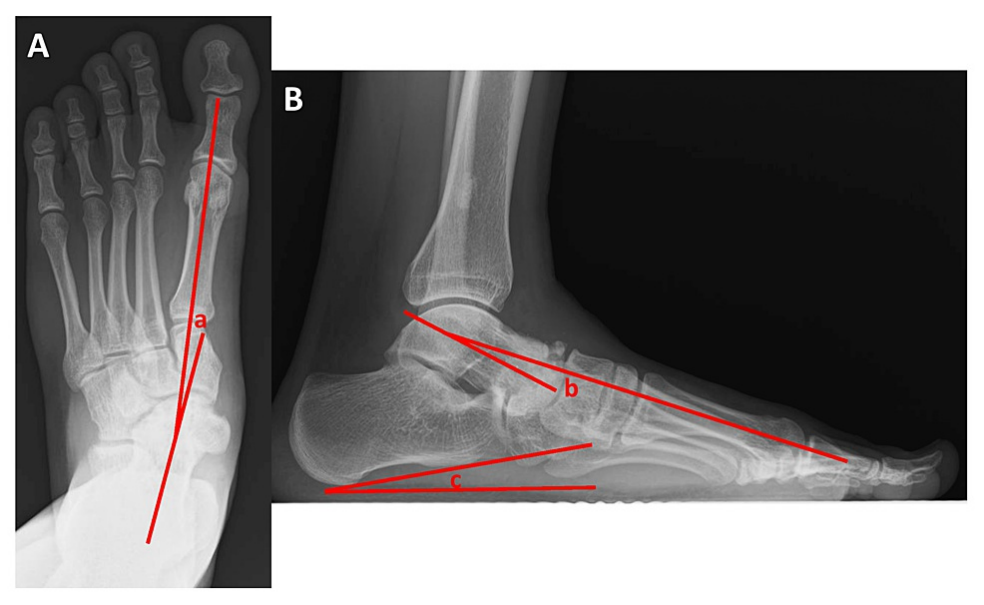
Plain radiograms with weight-bearing after the removal of the screw and compression staple at 13 months post-surgery. (A) Anteroposterior view. (B) Lateral view. a, b: talo-first metatarsal angle; c: calcaneal pitch

Thirteen months postoperatively, the JSSF midfoot scale score was 100 points, and all categories in the SAFE-Q also reached 100 points. The range of motion on the left side was as follows: dorsiflexion 25°; plantar flexion 45°; inversion 30°; and eversion 30°. His flat foot was also improved. In the anteroposterior view of the radiographs, the talo-first metatarsal angle improved to 7°. On lateral radiographs, the talo-first metatarsal angle improved to 8°, and the calcaneal pitch improved to 12° (Figure 4A-4B).

## Discussion

We report good clinical outcomes by talonavicular arthrodesis using a CCS and a compression staple in a patient with bipartite navicular bone. There are several advantages to using a combination of CCS and compression staples. Compression staples compress bone fragments through superelasticity and shape memory [9,10]. Compression staples can maximize the total contact area without multiple crossings of the fusion surface using screws [10,13]. In addition, compression staples are easy to insert and reduce operation time [14]. Especially for the dorsal aspect of the foot, a compression staple is less likely to cause implant-related symptoms than a locking plate because of its small size and low profile. Hoon et al. showed that staple fixations exhibited statistically higher compressive loads and contact areas across the osteotomy compared to plate fixations [10]. They also demonstrated that double staple fixations had the most consistent bending stiffness in all planes compared to single staple and plate fixations. Therefore, a CCS fixation was added to a single staple fixation in our case because one compression staple alone had a weak resistance to rotational and shear forces. As a result, bone union was achieved even in a poor bone condition of the talonavicular joint. The complicated shape of the talonavicular joint necessitates meticulous joint preparation for arthrodesis [1]. Previously, isolated talonavicular arthrodesis using screws [8] or a locking plate for the bipartite navicular bone was reported. Both locking plate or screw fixation for isolated talonavicular arthrodesis require sufficient space for the placement of screws for firm fixation. However, the small size of the navicular makes implant fixation difficult [15]. In addition, the talonavicular joint transfers the load between the talar head and the proximal articular surface of the navicular, and sliding, rolling, and rotational motions are used to control foot rigidity throughout the gait cycle [16]. High stress and complicated movement at the talonavicular joint negatively impact the fusion site of the talonavicular joint. The combination of a compression staple and CCS can yield good bone union with good clinical outcomes even in the poorly conditioned bipartite navicular bone.

Arthrodesis of the medial column is performed in the surgical treatment of bipartite navicular, although it decreases the range of motion of the midfoot. Internal fixation using screws with autologous cancellous bone grafting to restore midfoot motion and function has been reported to result in nonunion [3]. Therefore, arthrodesis, which involves several procedures, including fusion of the joint, is currently the most suitable surgical treatment. Wiley et al. reported that a good clinical outcome was obtained with triple arthrodesis for a bipartite navicular bone [1]. Reade et al. demonstrated that isolated talonavicular arthrodesis was performed for Müller-Weiss disease of a 25-year-old female, and fusion of the talonavicular joint was confirmed four months postoperatively [17]. Kitaura et al. showed that talonavicular arthrodesis and talonavicular-cuneiform arthrodesis were performed in two cases of Müller-Weiss disease; talonavicular-cuneiform arthrodesis achieved bone union, but talonavicular arthrodesis resulted in nonunion [18]. For patients with bipartite navicular bone, there may be no suitable treatment other than arthrodesis of the medial column. However, osteoarthritic changes in adjacent joints can be induced by sacrificing joint mobility. Tanaka et al. created a new crank-shaped joint between the talus and cuneiforms by the fusion of a medial fragment of the navicular to the talus and a lateral fragment of the navicular to the third cuneiform to preserve the partial range of motion of the talonavicular joint in a patient with bipartite navicular bone [19]. In our case, the decrease in range of motion was slight compared to that on the uninvolved side. This may be a compensatory movement of the naviculocuneiform joint. In the short term, isolated talonavicular arthrodesis does not interfere with daily life, including sports activities. However, osteoarthritis of adjacent joints may occur during long-term follow-up, although the incidence of adjacent joint disorder after isolated talonavicular arthrodesis is still unclear.

## Conclusions

Bipartita navicular bone is rare and has no established treatment. It caused foot pain, osteoarthritic changes around the navicular, and alignment abnormalities, which were improved with proper arthrodesis of the talonavicular joint. Among various fixation methods for fusion, a compression staple has unique advantages including providing persistent compression force and being easy to use. An isolated talonavicular arthrodesis using a combination of CCS and compression staple yielded good short-term clinical outcomes.
